# Electrospinning Chitosan/Fe-Mn Nanofibrous Composite for Efficient and Rapid Removal of Arsenite from Water

**DOI:** 10.3390/toxics12030230

**Published:** 2024-03-21

**Authors:** Lingli Min, Yahui Ma, Bi Zhang, Dulan He, Jinhua Chen, Xuerong Li, Shuhua Wang, Yulang Chi

**Affiliations:** 1 College of Resources and Environmental Science, Quanzhou Normal University, Quanzhou 362000, China; llmin@qztc.edu.cn (L.M.); shwang@qztc.edu.cn (S.W.); 2 College of Oceanology and Food Science, Quanzhou Normal University, Quanzhou 362000, China

**Keywords:** arsenite, chitosan, iron/manganese composite, nanofiber

## Abstract

Efficient removal of extremely mobile and toxic As(III) from water is a challenging but critical task. Herein, we developed a functionalized sorbent of chitosan nanofiber with iron–manganese (Fe-Mn@CS NF) using a one-step hybrid electrospinning approach to remove trace As(III) from water. Batch adsorption studies were performed to determine the adsorption efficiency under a variety of conditions, including contact time, starting concentration of As(III), ionic strength, and the presence of competing anions. The experimental results demonstrated that the concentration of As(III) dropped from 550 to less than 1.2 µg/L when using 0.5 g/L Fe-Mn@CS NF. This demonstrates the exceptional adsorption efficiency (99.8%) of Fe-Mn@CS NF for removing As(III) at pH 6.5. The kinetic tests revealed that the adsorption equilibrium was reached in 2.6 h, indicating a quick uptake of As(III). The ionic strength effect analysis showed that the adsorbed As(III) formed inner-sphere surface complexes with Fe-Mn@CS NF. The presence of SO_4_^2−^ or F^−^ had a negligible impact on As(III) uptake, while the presence of PO_4_^3−^ impeded As(III) absorption by competing for adsorption sites. The exhausted sorbent could be effectively regenerated with a dilute NaOH solution. Even after 10 cycles of regenerating Fe-Mn@CS NF, the adsorption efficiency of As(III) in natural groundwater was maintained over 65%. XPS and FTIR analyses show that the presence of M–OH and C–O groups on the sorbent surface is essential for removing As(III) from water. Overall, our study highlights the significant potential of Fe-Mn@CS NF for the efficient and quick elimination of As(III) from water.

## 1. Introduction

Arsenic (As) naturally exists in many rocks and sediments [1]. Human activities, such as mining, as well as the extensive use of this substance in industry, agriculture, and livestock, might lead to its release into groundwater [2,3]. Arsenic in drinking water sources has led to substantial health problems because of its toxicity, even at low levels [4,5]. Hence, a regulated maximum contamination limit (MCL) of 10 µg/L has been set for arsenic in drinking water by the World Health Organization (WHO) and the EPA of China. In natural waters, there are two types of inorganic arsenic: As(V) and As(III) according to different redox potentials. As(III) is highly hazardous and challenging to eliminate since it exists in an uncharged form at most pH levels [6].

In order to treat arsenic to a safe level, the chemical precipitation method is known as a cost-efficient approach. However, it is not successful for removing As(III). Ion exchange and reverse osmosis are more effective, but their operational and maintenance expenses are considerable, and they are prone to fouling. Adsorption is an appealing choice to accomplish this challenging task, particularly by facilitating the effective utilization of affordable biomaterials [3,7,8]. Chitosan is one of the most abundant natural biopolymers, because it can be produced cheaply from the deacetylation of chitin, a biopolymer that ranks second in abundance only to cellulose. The use of chitosan-based sorbents in water treatment has become an attractive choice due to their cost-effectiveness, non-toxicity, and biodegradability [9,10,11].

The adsorption properties of sorbents are primarily affected by their dimensions. When the dimension is reduced to the nanoscale scale, more functional atoms are exposed to the surface, reducing the adsorption equilibrium time significantly. Nanoparticle sorbents, for example, are known for their quick adsorption rates. However, they still have several drawbacks, including the tendency to aggregate into larger particles, difficulties in separating solids from liquids, and the risk of secondary contamination. Electrospinning technology is widely applied to prepare nanofibrous biomaterials. One advantage of electrospun nanofibers over nanoparticles is their ability to effectively sorb metal ions and arsenic from water [12,13,14] without the hassle of solid–liquid separation. Recently, a renewable polyethyleneimine/polyvinyl chloride nanofiber sorbent was prepared using the electrospinning technique [15]. The adsorption equilibrium was achieved after 90 min when the initial concentration of As(V) was 10 mg/L at a pH of 5.0. However, it is still challenging to remove trace As(III) efficiently, partly because As(III) exists as neutral molecules (H_3_AsO_3_) in groundwater [3].

Iron–manganese oxides are widely present in nature and have attracted significant interest for their exceptional ability to remove As(III) in water [16,17,18]. To the best of our knowledge, As(III) adsorbed onto chitosan nanofiber incorporating trace iron–manganese (<6%) has not been studied yet. The purpose of this work is to develop a cost-effective sorbent of iron–manganese-incorporated chitosan nanofiber (Fe-Mn@CS NF) for the efficient and quick elimination of As(III) from water. The physicochemical properties of the sorbent were explored by SEM, XRD, BET, FTIR, and XPS analyses. As(III) removal efficiency was evaluated by studies on both the kinetics and equilibrium adsorption isotherms. The influences of ionic strength and competitive anions on As(III) removal were further investigated. Furthermore, the As(III) desorption from Fe-Mn@CS NF by dilute NaOH solutions and the reusability of the sorbent after adsorption of As(III) in natural groundwater were explored.

## 2. Materials and Methods

### 2.1. Materials

Chitosan powder with an average molecular weight of 150,000 and poly (ethylene oxide) (PEO) particles with an average molecular weight of 1,000,000 were bought from Aladdin in Shanghai, China. FeCl_3_·6H_2_O, MnCl_2_, CH_3_COOH, NH_3_·H_2_O, NaOH, and HCl were also purchased from Aladdin. A stock solution containing 920 mg L^−1^ of arsenite was prepared by dissolving the correct quantity of NaAsO_2_ (SCRC, Shanghai, China) in ultrapure water and then adequately diluted before use. Other chemicals were purchased from Macklin (Shanghai, China). All chemicals used in this study were reagent-grade or better.

### 2.2. Preparation of Sorbents

Chitosan and PEO solutions at a concentration of 3.0% *w*/*v* were prepared separately by dissolving the polymers in acetic acid (50% *v*/*v*) and stirring with a magnetic stirrer until identical solutions were formed. The metal-free chitosan/PEO nanofiber (CS) was synthesized by combining chitosan and PEO in a ratio of 13.9:1 (*w*/*w*) to form the blend solution. A chitosan Fe-Mn hybrid solution was prepared by mixing chitosan, PEO, FeCl_3_·6H_2_O, and MnCl_2_ with acetic acid (50% *v*/*v*) and stirring the mixture for 6 h.

The design of the sorbents is based on our previous study on electrospun chitosan composite materials [12]. This previous study showed a positive correlation between the iron content added to chitosan and the adsorption performance of the material for inorganic arsenic. However, exceeding a metal dopant concentration of 4% in the sorbents for iron single element doping may result in drawbacks such as an unstable spinning solution or decreased spinning yield. Thus, on this study, we first set the iron amount at around 1.3% and fine-tuned the manganese element content. Once the appropriate quantity of manganese has been determined, we proceeded to further optimize the iron concentration.

Electrospinning experiments were conducted at an ambient temperature ranging from 23 to 25 °C with a relative air humidity between 33% and 40%. The electrospinning arrangement was detailed in our prior article [10]. The solution was expelled using a syringe flow pump at a consistent feed rate of 0.9 mL/h under an applied voltage of 17.5 kV. The distance between the needle tip and the collector was set at 13.5 cm. Nanofibers were produced on the collector’s surface after the acetic acid evaporated. The remaining solvent in the nanofibers was eliminated by drying in a dryer at 50 °C for 6 h. The nanofibers were crosslinked utilizing NH_3_·H_2_O, rinsed with DI water to eliminate PEO, and finally dried for batch adsorption tests.

### 2.3. Characterization

The surface features of the nanofibers were examined by SEM, Hitachi SU 8020, Chiyoda City, Japan. The samples were seen using a working distance of 7.4 mm and an accelerating voltage of 3 kV. The X-ray diffraction (XRD) analysis was conducted using a Y-2000 X-ray diffractometer from Dandong (China) Aolong Radiative Instrument Co., Ltd. (Dandong, China). The current in operation was 20 mA, while the voltage was 40 kV. Scans were conducted from 5° to 90° at a rate of 3° per minute, with an increment of 0.02°.

X-ray photoelectron spectroscopy (XPS) spectra of the sorbent were obtained before and after arsenic absorption using the Thermo ESCALAB 250XI spectrometer from Thermo Fisher Scientific, Waltham, MA, USA, with 150 W monochromatized Al Ka radiation (1486.6 eV). The wide-scan XPS spectra were obtained using an energy range of 0–1350 eV with a step size of 1.0 eV. The high-resolution XPS scans were performed using a 0.1 eV step size specific to the peak under examination. The XPS data were obtained as binding energies and analyzed using nonlinear least squares curve fitting software. The C1s electron binding energy of 284.8 eV for graphitic carbon was utilized as the calibration reference.

### 2.4. Adsorption Tests

Each As(III) solution of the required concentration was combined with the sorbent in a 50 mL conical flask. Following agitation for a set duration at 298 K, the equilibrium As(III) solution was passed through 0.45 μm filter membranes and subsequently analyzed by ICP-MS (Agilent 7500cx, Santa Clara, CA, USA). Kinetics tests were performed by introducing the sorbent at a concentration of 0.45 g/L into a 110 mL solution containing As(III). The samples were collected at suitable time intervals and examined for As(III) levels using ICP-MS. Isotherm experiments were carried out by altering the initial As(III) concentrations (ranging from 6.9 to 2300 µg/L) while maintaining a constant sorbent dose of 0.28 g/L. As(III) concentrations were evaluated after 24 h of interaction time. The selected anions for competition are SO_4_^2−^, CO_3_^2−^, SiO_3_^2−^, PO_4_^3−^, and F^−^. The concentrations of these anions ranged from 0 to 0.5 mM, respectively.

### 2.5. Regeneration Tests

For the sorption test, 0.015 g of Fe-Mn@CS NF was added to a bottle containing a 1000 µg/L As(III) solution. After stirring for 12 h, the sorbent was separated and retrieved from the solution. The residual arsenic concentration was analyzed by ICP-MS. The desorption test involved adding the As(III)-containing sorbent to a 20 mL NaOH solution with concentrations varying from 2 mM to 250 mM. The mixture was stirred for 20 min, and then the regenerated sorbent was isolated from the NaOH solution. Once washed and dried, the regenerated sorbent was used in the next sorption–desorption process.

Furthermore, to guarantee the regenerability of Fe-Mn@CS NF, ten cycles of adsorption and desorption were carried out using raw groundwater spiked with 0.12 mg/L of As(III). The groundwater samples were collected near Fuxi Road in Quanzhou, China. Water quality data are contained in Table S1. A 50 mM NaOH solution was chosen for the regeneration tests. After desorption, the sorbent was washed repeatedly until the effluent achieved a pH of around 7.0 before starting the next adsorption process. The regeneration tests were repeated ten times.

## 3. Results and Discussion

### 3.1. Optimization of Fe-Mn@CS NF

**Figure 1:**
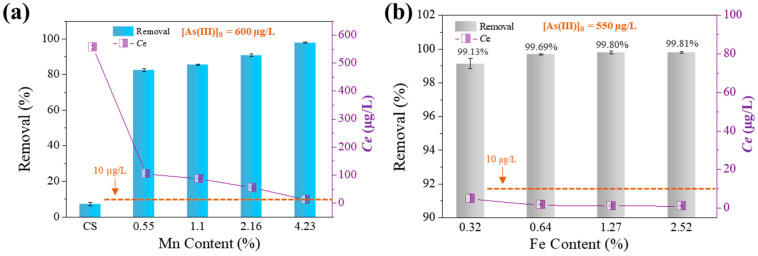
Effect of Mn content (**a**) and Fe content (**b**) on As(III) adsorption and the residual As(III) in water after adsorption. Solid dosage = 0.425–0.5 g/L, initial pH = 6.5, 150 r/min for 24 h, T = 25 °C.

This work investigated chitosan-based nanofibers containing iron and manganese with varying Fe-Mn contents. Out of the four tested sorbents with Mn doping (each with a set Fe content of 1.27%) and one metal-free sorbent (CS, as shown in Figure 1a), CS was found to be ineffective, with a removal rate of 7.24%. Nevertheless, the incorporation of a small proportion of metals (1.82–5.5%) greatly enhances the effectiveness of pure chitosan nanofiber membranes in eliminating arsenic from water. This finding agrees with what we found in our earlier study [12]. More metal doping means more active sites for absorption on the surface of the adsorbent because the doped metal elements are spread equally over the surface and pores of the material [19]. The Mn-doped sorbent with a 4.23% concentration showed the highest performance in removing As(III), with a removal rate of 97.98%. Moreover, the increased proportion of Mn led to a decreased level of residual As(III) (Figure 1a). The findings indicate that the electrospinning technique is a cost-effective and straightforward way for fabricating composite nanomaterials employed in the removal of trace levels of contaminants from water.

Figure 1b shows a slow rise in the removal rate of As(III) by the sorbent (each with a Mn content of 4.23%) as the iron content rises, ranging from 99.13% to 99.81%. The concentration of high-As(III) water (550 µg/L) can be reduced to levels that meet the world drinking standard, which is less than 1.2 µg/L, by utilizing only 0.5 g/L sorbent, as shown in Figure 1b. As expected, the quantity of possible active sorption sites on the sorbent surface rises with the Fe-Mn levels in the composites, resulting in an enhanced capacity for sorbing As(III). The findings on the affinity of As(III) for Fe-Mn-loaded sorbents are consistent with previous research [9]. The chitosan nanofiber containing 4.23% Mn and 1.27% Fe exhibited the highest electrospinning yield and an exceptional As(III) removal rate of 99.80%. Consequently, it was selected for subsequent studies.

### 3.2. Characterization of CTS/Fe-Mn NF

**Figure 2:**
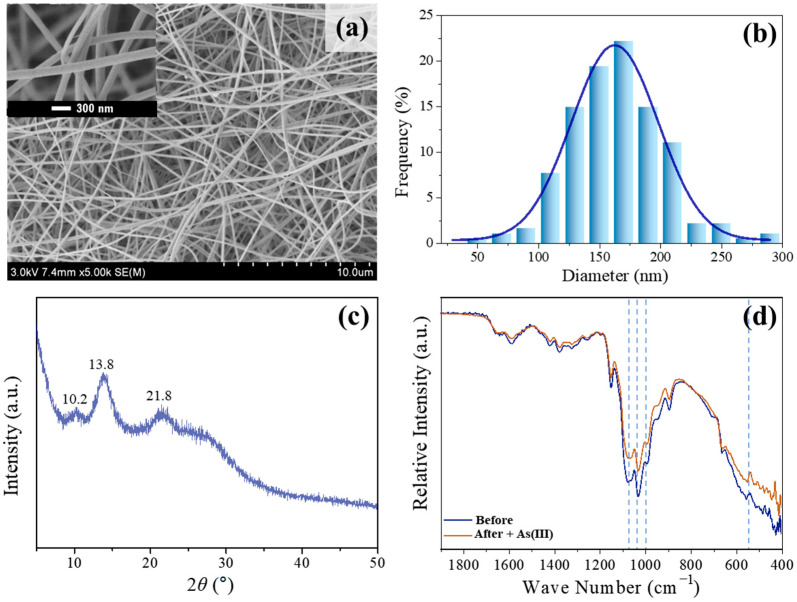
SEM pictures (**a**), dispersion of nanofiber diameters (**b**), and XRD pattern (**c**) of electrospinning Fe-Mn@CS composite. (**d**) FTIR spectra of the fresh sorbent and the As(III) loaded Fe-Mn@CS NF.

Figure 2a,b display the SEM pictures and the distribution of fiber diameters for the electrospinning Fe-Mn@CS composite. The nanofibrous membrane exhibits a homogeneous and smooth surface, devoid of any beads. The average diameter of the fibers is 160 nm. The nanofibrous composite exhibited a BET surface area of 7.24 m^2^/g. Figure 2c displays the X-ray diffraction spectra of Fe-Mn@CS NF. The spectra display three distinct peaks at 2*θ* angles of 10.2°, 13.8°, and 21.8°, which suggest that the Fe-Mn elements in Fe-Mn@CS NF are in an amorphous state. Amorphous sorbents could increase the specific surface area, which is advantageous for adsorption [20,21,22].

Many chitosan-incorporating metal sorbents typically feature metal hydroxyl groups (M–OH) on their surfaces; these are the most prevalent and effective adsorption sites for the removal of anionic pollutants from water. The active adsorption sites in Fe-Mn@CS NF were identified using FTIR analysis. Figure 2d displays the FTIR spectra of the sorbent before and after the interaction with As(III). Three peaks at 1073, 1029, and 990 cm^−1^ are due to the bending vibration of the hydroxyl group (M–OH) [23]. Extensive studies have demonstrated that surface hydroxyl groups have a strong affinity for ligands like As(III) and As(V) [8,9,24]. Following the adsorption of As(III) in our investigation, there was a notable decrease in the intensity of the peaks observed at 1073, 1029, and 990 cm^−1^. Additionally, the M–O stretching vibration at 542.8 cm^−1^ [25] was no longer observed after As(III) adsorption. These findings indicate that the M–OH group played a predominant role in the adsorption reaction.

### 3.3. Adsorption Kinetics and Isotherms

**Figure 3:**
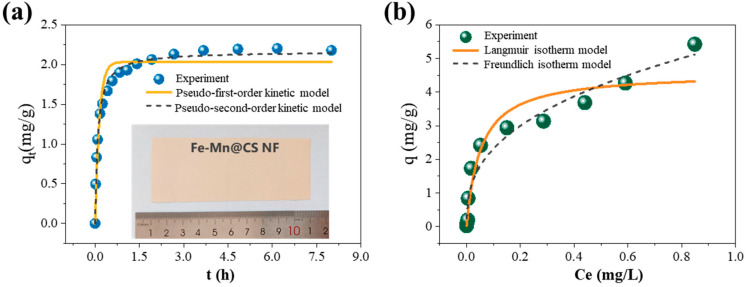
Kinetic adsorption data and corresponding mathematical models (**a**) and adsorption isotherms (**b**) of As(III) on Fe-Mn@CS NF. The lower part of Figure 3a displays an image of the sorbent. Solid dosage = 0.5 g/L, initial pH = 6.5, 150 r/min for 24 h, T = 25 °C.

To acquire kinetic information regarding the adsorption of As(III), bulk adsorption experiments were conducted with a time variation of 8 h and a natural pH of 6.5. The quantity of As(III) that adsorbed onto Fe-Mn@CS NF over time is depicted in Figure 3a. The adsorption process is characterized by a rapid initial uptake within the first 50 min, followed by a slower rate of absorption until equilibrium is reached after 2.6 h. Based on the observed short adsorption equilibrium time and high initial absorption rate, it can be concluded that the synthesized nanofiber sorbent possesses a substantial number of active sites that are accessible for the adsorption of As(III).

The kinetic data were analyzed using pseudo-first-order and pseudo-second-order models. Table 1 displays the kinetic parameters for the two models. The correlation coefficients showed that the pseudo-second-order model (R^2^ = 0.988) was better than the pseudo-first-order model (R^2^ = 0.936). This suggests that As(III) may have been chemically bonded to the surfaces through the creation of links resulting from the exchange of electrons between the adsorbent and the adsorbate [26].

The As(III) adsorption isotherms at different initial concentrations are depicted in Figure 3b. The equilibrium of sorption is typically characterized by an isotherm equation, in which the parameters represent the surface characteristics and affinity of the sorbent. The Langmuir and Freundlich equations are frequently utilized to quantitatively explain isothermal models, represented by Equations (1) and (2) correspondingly.

(1) $$q_{e}=\frac{q_{\max}{\;b\;C}_{e}}{1+{b\;C}_{e}},$$

(2) $$q_{e}=K_{F}C_{e}^{1/n},$$

The variables q_max_ (mg/g) and b (L/mg) represent the maximum quantity of arsenic adsorbed per unit weight of the sorbent and the adsorption affinity of the binding sites, respectively, as measured by the Langmuir constant. Ce (mg/L) represents the equilibrium concentration of As(III). Denoting the Freundlich constants are the symbols K_F_ and n. Typically, the adsorption of a single layer of adsorbate onto a uniform surface of the sorbent is described by the Langmuir equation, under the assumption that there is no intermolecular interaction among the adsorbate molecules. Conversely, the Freundlich equation is frequently utilized to characterize the process of adsorbate layer adsorption onto a heterogeneous surface. In addition, Freundlich parameters tend to produce isotherms that are more closely related to experimental results at lower concentrations, while Langmuir isotherms tend to give a better fit for the adsorbate at higher concentrations [27,28].

The constants obtained for the isotherm models are detailed in Table 2. At a maximum concentration of Ce equal to 0.85 mg/L, the calculated maximum adsorption capacity (q_max_) is 4.59 mg/g. The obtained q_max_ value exceeds the recently reported low-cost sorbents such as the seed pods biosorbents [8] and the iron-coated cork granulates [29]. The higher coefficients for the Freundlich isotherm (R^2^ = 0.955) show that the Freundlich model is better suited for predicting the absorption behavior. The fact that the adsorption reaction takes place in a system with a low adsorbate concentration is likely the primary cause of this result [27]. This suggests that the multilayer adsorption process is likely to be preferred [28].

Table 3 outlines the adsorption properties of several sorbents as documented in the literature, including the testing circumstances. The data suggest that Fe-Mn@CS NF attains equilibrium more rapidly than the alternative sorbents, indicating that it may serve as a viable and effective sorbent for the remediation of groundwater contaminated with trace amounts of As(III).

### 3.4. Effects of Iron Strength and Common Substances

**Figure 4:**
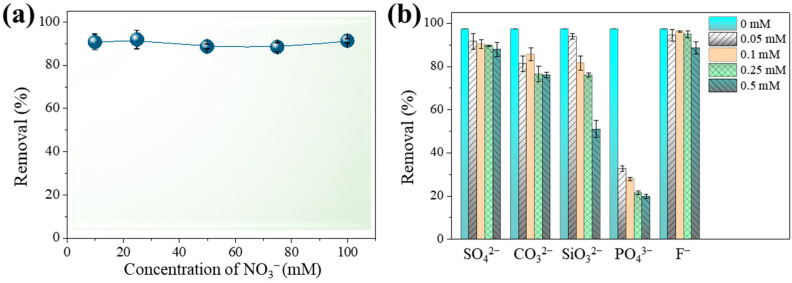
Effect of ionic strength (**a**) and typical anions (**b**) on the efficiency of As(III) removal by Fe-Mn@CS NF. Solid dosage = 0.5 g/L, [As(III)]_0_ = 1.3 mg/L, initial pH = 6.5, 150 r/min for 24 h, T = 25 °C.

Adsorption mechanisms involving the adsorbates and the sorbents may be categorized as inner- and outer-sphere complexes [33,34]. Arsenic adsorption for outer-sphere surface complexes is predicted to decrease as ionic strength increases, whereas arsenic adsorption for inner-sphere surface complexes is neither changed nor increased [34]. Figure 4a shows that elimination rates were kept consistently over 88.3% when the concentration of NO_3_^−^ was increased from 10 to 100 mM, indicating that inner-sphere complexation was the leading mechanism in As(III) sorption.

The presence of co-ions in natural waters might impede the adsorption process by engaging in competition for or obstructing adsorption sites on the sorbent’s surface. Figure 4b depicts the effects of several anions on As(III) sorption on Fe-Mn@CS NF. The coexistence of SO_4_^2−^ or F^−^ even at 0.5 mM hardly affected As(III) sorption on the sorbent. In contrast to arsenic, which was adsorbed through inner-sphere complexation, SO_4_^2−^ was mostly adsorbed through outer-sphere complexation at a pH over 6 [35], having no impact on As(III) sorption. The coexistence of CO_3_^2−^ slightly reduced the sorption of As(III) (Figure 4b). CO_3_^2−^ has a minor impact since its affinity for sorbents is generally lower than that of arsenic [36]. SiO_3_^2−^ and PO_4_^3−^ may hinder arsenic adsorption through the formation of inner-sphere complexes. Figure 4b shows that the occurrence of SiO_3_^2−^ slightly lowers As(III) removal efficiency. A similar phenomenon was reported by Wei et al. [37]. Comparatively, 0.05 mM PO_4_^3−^ suppressed As(III) adsorption by 64%. The adverse impact of PO_4_^3−^ may result from competing for functional sites with arsenic [38,39], since As and P are chemical analogues and they have similar chemical behaviors in water. These findings provide further evidence that As(III) adsorption onto Fe-Mn@CS NF occurs via an inner-sphere complex process.

### 3.5. Regeneration Tests

**Figure 5:**
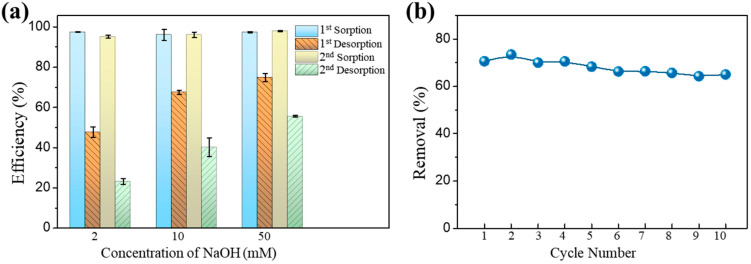
(**a**) Regeneration of Fe-Mn@CS NF with different concentrations of NaOH. Solid dosage = 0.75 g/L, [As(III)]_0_ = 1.0 mg/L, initial pH = 6.5, 150 r/min for 24 h, T = 25 °C. (**b**) Cyclic adsorption–regeneration runs of the Fe-Mn@CS NF by 50 mM NaOH toward actual groundwater spiked with 0.12 mg As(III)/L.

Reusability and regeneration may be regarded as the most essential qualities of a prospective sorbent. Among these, sorbent regeneration stands out as a pivotal stage that substantially impacts both process expenses and the retrieval of pollutants. Sodium hydroxide (NaOH) is commonly employed as a basic solution to extract arsenic species from chitosan [12,40]. This study examined different concentrations of NaOH to determine the optimal concentration for regeneration. The reusability of Fe-Mn@CS NF was tested through two sorption–desorption cycles (Figure 5a). After regenerating the sorbent with different concentrations of NaOH solutions, the adsorption efficiency for As(III) remained above 95.1%. However, the most effective desorbent, a 50 mM NaOH solution, only removed 74.9% and 55.6% of the adsorbed As(III) in the first and second cycles, respectively. When dealing with groundwater that has been spiked with As(III) and contains substantial quantities of other ions, the Fe-Mn@CS NF demonstrated adsorption efficiencies of over 64% even after undergoing 10 cycles of regeneration (Figure 5b). These above observations suggested that Fe-Mn@CS NF has good mechanical characteristics and reusability, making it a viable and sustainable adsorbent for removing As(III) from aqueous media, even though the low-concentration alkaline solution desorption system still needs further research and improvement. For instance, the desorption rate of the adsorbent might be enhanced by developing a multi-component desorption system or increasing the concentration of the NaOH solution [41].

### 3.6. Mechanism Study

**Figure 6:**
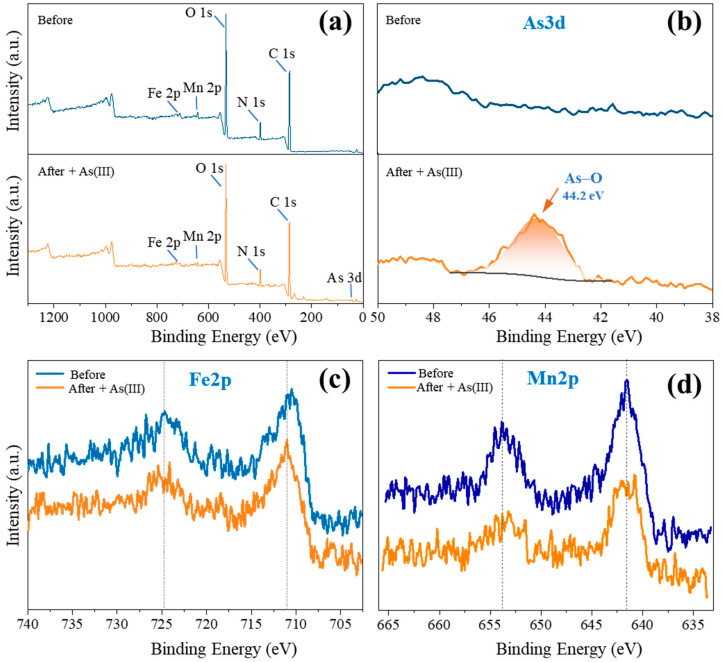
XPS spectra of wide scan (**a**), As3d (**b**), Fe2p (**c**), and Mn2p (**d**) of the fresh sorbent and the As(III)-loaded Fe-Mn@CS NF.

XPS analysis was conducted in order to identify the functional groups of the Fe-Mn@CS NF and examine their interactions with As(III) throughout the adsorption procedure. Figure 6 depicts the XPS spectra of the unloaded and As(III)-loaded sorbent. After reacting with As(III), the As3d core level emerged, suggesting the existence of arsenic on the surface of Fe-Mn@CS NF (Figure 6a,b). The binding energy of As3d was 44.2 eV, falling within the typical range of 44.3–44.5 eV for As(III) in arsenic oxides [16,23]. Figure 6c,d show the Fe2p and Mn2p spectra of the sorbent prior to and subsequent to reacting with As(III). Fe(III) was the oxidation state of iron species present on the Fe-Mn@CS NF surfaces, as shown by the peak shape and the binding energy of around 711.0 eV (Figure 6c) [16,23,25]. There was no obvious change in Fe2p spectrum after the reaction with As(III). However, the peak of Mn2p at 640.2 eV shifted up to 640.4 eV, and the peak at 652.4 eV shifted down to 651.7 eV after the adsorption of As(III) (Figure 6d), demonstrating significant interactions between As(III) and Mn elements.

**Figure 7:**
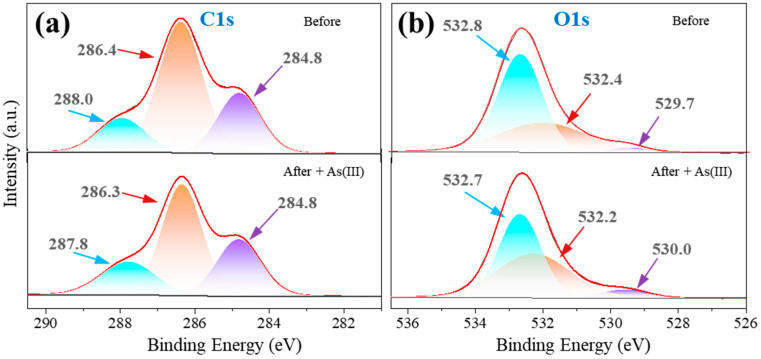
XPS spectra of C1s (**a**) and O1s (**b**) of the fresh sorbent and the As(III)-loaded Fe-Mn@CS NF.

The C1s spectra were analyzed to understand the structural changes related to As(III) adsorption. It was separated into three peaks at 284.8 eV, 286.3 eV, and 287.8 eV, corresponding to the C–C/C–H, C–O/C–N, and C=O/O–C–O groups, respectively (Figure 7a and Table S2). The proportion of the peak at 286.3 eV (related to the C–O/C–N group) reduced from 57.6% to 51.3% after As(III) adsorption, suggesting the significant involvement of the C–O/C–N group on the sorbent surface in As(III) uptake.

The O1s spectra of Fe-Mn@CS NF before a reaction were identified into three peaks at 529.7, 532.4, and 532.8 eV, respectively (Figure 7b). The peak at 529.7 eV corresponds to the lattice oxygen of M–O (M = Fe and Mn). Its fraction increased from 5.3% to 6.3% following the reaction, indicating the creation of M–O–As (M = Fe or Mn) [42,43]. The peak at 532.4 eV in the O1s spectrum corresponds to the –OH group, and its relative amount reduced from 46.0% to 37.8% post-reaction. The results indicated that As(III) replaced the hydrogen ions in –OH to create As–O groups, leading to a reduction in hydroxyl amount.

To summarize, the FTIR (Figure 2d) and XPS analyses suggest that the Fe-Mn@CS NF contains a substantial number of –OH groups on its surface. These groups are highly efficient in the removal of As(III) from water. This has the potential to enhance the preferred attachment of ligands, particularly Lewis bases like arsenites, by forming inner-sphere complexes.

## 4. Conclusions

Efficient removal of highly mobile and poisonous As(III) from water is a difficult task, yet it is of utmost importance. In this work, a novel nanoscale sorbent of chitosan iron–manganese composites (Fe-Mn@CS NF) was successfully fabricated via one-step hybrid electrospinning. The composite offered a plentiful supply of hydroxyl groups (–OH) for the purpose of removing arsenic. This enhances the possibility of ligand exchange, resulting in the creation of inner-sphere complexes with As(III). The experimental data indicated that the concentration of As(III) decreased from 550 µg/L to levels far below the allowable limit for drinking water (<1.2 µg/L). The adsorption process reached equilibrium quickly in 2.6 h, displaying chemisorption characteristics and a good fit with the pseudo-second-order model (R^2^ = 0.988). PO_4_^3−^ hindered the removal of As(III) by competing for adsorption sites, while SO_4_^2−^ and F^−^ had little impact on the absorption of As(III). The exhausted sorbent was successfully regenerated with 50 mM NaOH. In addition, the Fe-Mn@CS NF maintained adsorption efficiencies of over 64% for As(III)-spiked groundwater even after 10 cycles of regeneration. As(III) is adsorbed onto Fe-Mn@CS NF through the formation of inner-sphere surface complexes with As–O–M (M = Fe or Mn) linkages. To address the needs for both irrigation and drinking water, we propose using this functional sorbent either as a solid filler or in conjunction with a biofilm reactor for the deep treatment of surface or groundwater. This work concludes that electrospinning is a simple, inexpensive, and effective way to create composite nanomaterials for deep water purification of trace contaminants, and it introduces a nanofibrous sorbent that is both highly efficient and environmentally friendly for removing As(III) from water.

## Figures and Tables

**Table 1:** Parameters for the pseudo-first-order and pseudo-second-order kinetic models.

Pseudo-First-Order, q_t_ = q_e_(1 − e^−k^_1_^t^)			Pseudo-Second-Order, q_t_ = k_2_q_e_^2^t/(1 + k_2_q_e_t)		
q_e_ (mg/g)	k_1_ (h^−1^)	R^2^	q_e_ (mg/g)	k_2_ (g/(mg·h)	R^2^
2.03	7.24	0.936	2.17	4.98	0.988

**Table 2:** Parameters for the Langmuir and Freundlich isotherm models.

Langmuir			Freundlich		
q_max_ (mg/g)	b (L/mg)	R^2^	K_F_ ((mg/g)(mg/L)^n^)	1/n	R^2^
4.59	18.97	0.924	5.44	0.37	0.955

**Table 3:** Comparison of sorbents for As(III) removal.

Sorbents	pH	[As(III)]_0_ (mg/L)	Temperature (°C)	Equilibrium Time (h)	Ref.
Fe_2_O_3_ nanoclusters	9	3	25	6	[25]
Fe-Mn NPs	5	10	25	24	[30]
Fe-Zr@AC	7	1.5	25	6	[31]
CTS-Fe-Mn	/	/	25	20	[9]
Fe-Mn-Al oxides	/	1.98	26	8	[32]
Fe-Mn@CS NF	6.5	1.2	25	2.6	This study

## Data Availability

Data will be made available on request.

## Supplementary Materials

The following supporting information can be downloaded at: https://www.mdpi.com/article/10.3390/toxics12030230/s1, Table S1: Concentration of the dissolved ions in the natural groundwater sample. Table S2: Binding energies and relative contents of C and O in the fresh and As(III)-loaded biosorbent.
