# Supplementary material for: Electrospinning Chitosan/Fe-Mn Nanofibrous Composite for Efficient and Rapid Removal of Arsenite from Water
Source: Toxics. 2024 Mar 21;12(3):230. doi: 10.3390/toxics12030230 (PMC10974202; doi:10.3390/toxics12030230)
Supplement: Supplementary file 1 [file toxics-12-00230-s001.zip › toxics-2902942-supplementary.pdf]

Electrospinning Chitosan/Fe-Mn Nanofibrous Composite for Efficient and Rapid Removal of Arsenite from Water

Lingli Min [1], Yahui Ma [1], Bi Zhang [1], Dulan He [1], Jinhua Chen [1], Xuerong Li [1], Shuhua Wang [1] and Yulang Chi [2,*]

[1] College of Resources and Environmental Science, Quanzhou Normal University, Quanzhou 362000, China

[2] College of Oceanology and Food Science, Quanzhou Normal University, Quanzhou 362000, China

* Corresponding author. College of Oceanology and Food Science, Quanzhou Normal University, 398 Donghai Road, Quanzhou 362000, China

E-mail address: ylchi@qztc.edu.cn (Y. Chi).

Supplementary data

Table S1. Concentration of the dissolved ions in the natural groundwater sample. (Unit: mg/L)

**As**	**Fe**	**Cu**	**Zn**	**Cr**	**Pb**
0.00035	0.00112	0.00012	0.00186	0.00011	0.00033
F	Cl^-	SO_4^{2-}	NO_3^-	PO_4^{3-}	F^-
0.517	53.4	69.5	12.1	0.065	0.517

Table S2. Binding energies and relative contents of C and O in the fresh and As(III)-loaded biosorbent.

**Valence state**	**Sample**	**Assignment**	**Binding energy (eV)**	**Relative Content (%)**
C 1s	Fe-Mn@CS	C–C/C–H	284.8	26.9%
		C–O/C–N	286.4	57.6%
		C=O/O–C–O	288.0	15.5%
	Fe-Mn@CS-As	C–C/C–H	284.8	28.8%
		C–O/C–N	286.3	51.3%
		C=O/O–C–O	287.8	19.9%
O 1s	Fe-Mn@CS	M–O	529.7	5.3%
		–OH	532.4	46.0%
		H_2O	532.8	48.7%
	Fe-Mn@CS-As	M–O	530.0	6.3%
		–OH	532.2	37.8%
		H_2O	532.7	55.9%
